# Aortic Hemodynamics with Accelerated Dual-Venc 4D Flow MRI in Type B Aortic Dissection

**DOI:** 10.3390/app13106202

**Published:** 2023-05-18

**Authors:** Ozden Kilinc, Justin Baraboo, Joshua Engel, Daniel Giese, Ning Jin, Elizabeth K. Weiss, Anthony Maroun, Kelvin Chow, Xiaoming Bi, Rachel Davids, Christopher Mehta, S. Chris Malaisrie, Andrew Hoel, James Carr, Michael Markl, Bradley D. Allen

**Affiliations:** 1 Department of Radiology, Northwestern University, Chicago, IL 60611, USA; 2 Department of Biomedical Engineering, Northwestern University, Chicago, IL 60611, USA; 3 Magnetic Resonance, Siemens Healthcare GmbH, 91052 Erlangen, Germany; 4 Institute of Radiology, University Hospital Erlangen, Friedrich-Alexander-Universität Erlangen-Nürnberg (FAU), 91054 Erlangen, Germany; 5 Cardiovascular MR R&D, Siemens Medical Solutions USA, Inc., Cleveland, OH 44139, USA; 6 Cardiovascular MR R&D, Siemens Medical Solutions USA, Inc., Chicago, IL 60611, USA; 7 Department of Surgery (Cardiac Surgery), Northwestern University, Chicago, IL 60611, USA; 8 Department of Surgery (Vascular Surgery), Northwestern University, Chicago, IL 60611, USA

**Keywords:** dissection, 4D flow MRI, flow, aorta, type B aortic dissection, imaging, quantitative imaging

## Abstract

The aim of this study is to investigate the applicability of the dual-venc (DV) 4D flow magnetic resonance imaging (MRI) to quantify the complex flow patterns in type B aortic dissection (TBAD). One GRAPPA-accelerated single-venc (SV) and one compressed-sensing (CS) accelerated DV 4D flow MRI sequences are used to scan all subjects, including twelve chronic TBAD patients and two volunteers. The scans are performed twice for the reproducibility assessment of the scan protocols. Voxelwise quantitative flow parameters including kinetic energy (KE), peak velocity (PV), forward and reverse flows (FF, RF) and stasis are calculated. High-venc (HV) data from the DV acquisition are separately analyzed. The scan time reduction by the CS-accelerated DV 4D flow MRI acquisition is 46.4% compared with the SV acquisition. The DV velocity-to-noise ratio (VNR) is higher compared with HV (*p* = 0.000). No true lumen (TL) parameter shows a significant difference among the acquisition types (*p* > 0.05). The false lumen (FL) RF is higher in SV compared with the DV acquisition (*p* = 0.009). The KE is higher (*p* = 0.038) and stasis is lower (*p* = 0.01) in HV compared with SV acquisition. All FL parameters except stasis are higher and stasis is lower in HV compared with DV acquisition (*p* < 0.05). Positive Pearson correlations among the acquisition types in TL and high agreements between the two scans for all acquisition types are observed except HV RF in the FL, which demonstrates a moderate agreement. The CS-accelerated DV 4D flow MRI may have utility in the clinical daily routine with shortened scan times and improved velocity measurements while providing high VNR in TBAD. The observed hemodynamic flow trends are similar between GRAPPA-accelerated SV and CS-accelerated DV 4D flow MRI acquisitions; however, parameters are more impacted by CS-accelerated HV protocol in FL, which may be secondary to the CS regularization effects.

## 1. Introduction

Aortic dissection is a serious vascular injury where high blood pressure flow between the layers of the aorta caused by an intimal tear results in the formation of true (TL) and false lumen (FL) separated by an intimal flap [1–3]. Stanford type A aortic dissection (TAAD) occurs in the ascending aorta, whereas Stanford type B aortic dissection (TBAD) originates distal to the left subclavian artery and extends into the descending aorta [4]. Descending aorta dissections may occur in isolation (de novo TBAD [dnTBAD]), or secondary to the TAAD repairs, where patients may have chronic residual descending aorta dissection (rTAAD) after the surgical procedure for TAAD. TBAD can be medically managed with anti-impulse therapy if there are no signs of complicated dissection such as end-organ ischemia or rupture [2,4–7]. Even though the anti-impulse therapy is an effective strategy for stable TBAD treatment, a significant percentage of these patients need surgical intervention during the follow-up period secondary to the progressive FL expansion and therefore increased risk of aortic rupture [1,2]. Rapid diagnostic imaging with computed tomography angiography (CTA) or magnetic resonance angiography (MRA) is crucial for the clinical management of TBAD; however, traditional image-based management and risk-stratification have been primarily based on morphologic features of the aorta [8].

Time-resolved 3D phase contrast magnetic resonance imaging (MRI) with 3-directional velocity encoding (4D flow MRI) has been a widely accepted, useful investigational tool in evaluating several cardiovascular pathologies, including aortic dissection [1,5,9–13]. In vivo blood-flow characterization with 4D flow MRI has potential utility in identifying TBAD patients with enlarging aortas by the quantitative flow pattern assessment at the entry tear and in the FL [1,6,9–19]. Despite its benefits, long scan times associated with the multidimensional imaging and single velocity encoding (venc) level potentially limit the clinical adoption of the traditional 4D flow MRI. There are several potential consequences of single-venc (SV) 4D flow MRI acquisitions as velocity noise is directly proportional to the venc. The velocity (v) aliasing may occur for unpredictable high blood flow velocities (v > venc) such as in TL in TBAD and additionally, increased noise for slow flow regions (v < venc) may also be observed such as in FL in TBAD.

The venc used in the SV 4D flow MRI acquisitions is usually adjusted to the estimated peak velocity (PV) to avoid velocity aliasing, limiting the evaluation of the slow flow velocities [20–22]. However, there is a direct positive correlation between the velocity-noise-ratio (VNR) and the measured velocity and inverse relationship between the VNR and the venc; consequentially, the higher the venc, the lower VNR. This is especially important in regions with a slow flow such as in FL in TBAD [23]. Ideal hemodynamic evaluation of cardiovascular diseases should be performed by a technique that maintains high VNR across the encountered range of velocities. However, this range is very broad in TBAD as there are large differences in TL and FL velocities. It is also likely that poor VNR will lead to reduced accuracy of more advanced hemodynamic parameters such as flow stasis and kinetic energy (KE) which may be important markers of adverse outcome risk in TBAD [18]. To address this problem, low-venc (LV) and high-venc (HV) images are being acquired in the same scan with dual-velocity encoded (dual-venc—DV) 4D flow MRI using methods such as Bayesian analysis where the aliased data can be recovered using the HV data while preserving the favorable VNR of the LV data [24–26]. Combining DV 4D flow MRI method with compressed sensing (CS) acceleration technique may provide additional critically important benefits for TBAD management such as shorter scan times, as CS can significantly accelerate MRI acquisitions utilizing the inherent sparsity of MRI data [27–30].

In our previous study, we investigated the applicability of the CS-accelerated SV 4D flow MRI in TBAD with no impact by the DV methodology [31]. Here, in this study, we aim to systematically evaluate the potential utility of CS-accelerated DV 4D flow MRI with an acceleration level of 7.7 (R = 7.7) in aortic hemodynamics, including KE, PV, forward flow (FF), reverse flow (RF) and flow stasis in a cohort of chronic TBAD patients and volunteers. We have provided the detailed comparisons of the quantitative flow parameters in TBAD between the SV GRAPPA-accelerated 4D flow MRI and CS-accelerated DV 4D flow MRI acquisitions and discuss our results, taking into account the results from the previously published studies available in the literature. We hypothesize that DV 4D flow MRI acquisition improves the characterization of flow hemodynamics relative to SV 4D flow MRI acquisition by better capturing the full dynamic range of velocities in TBAD while maintaining a high VNR.

## 2. Materials and Methods

### Study Cohort

This study was approved by the Institutional Review Board and written informed consent was obtained from all subjects. As described in our previous study [31], the prospectively recruited cohort included twelve type B aortic dissection (TBAD) patients (57.75 ± 7.04 years old; 5-female, 7-male) including 6 medically managed de novo TBAD (dnTBAD) and 6 residual descending aorta dissection (rTAAD) cases and two healthy volunteers (a 28-year-old male and a 21-year-old female). The scans were performed for all subjects twice with the same scan protocol 5 to 10 days apart to evaluate the reliability of the acquisitions. The overall cohort included both the baseline and follow-up scans to be used for the groupwise comparisons of the quantitative hemodynamic parameters and for the correlations among the acquisition types. The volunteer data (entire aorta) was added to the true lumen (TL) analysis of the patients whereas the false lumen (FL) analysis was performed in the patient group only.

### Image Acquisition

All 4D flow magnetic resonance imaging (MRI) scans were performed on a 1.5T MRI system (MAGNETOM Sola, Siemens Healthcare, Erlangen, Germany). The volumetric coverage of the whole heart and entire aorta during the free-breathing non-contrast scan was provided and retrospective ECG gating was used. Respiratory navigator gating was used only for sagittally acquired dual-venc (DV) acquisitions as single-venc (SV) scans were acquired in coronal orientation with phase encoding left to right, which is less sensitive to respiratory motion artifacts. The DV scans were sagittally performed to reduce the scan time, as coronal 4D flow MRI scans take longer time. The scan protocol included one conventional GRAPPA-accelerated (R = 2) acquisition SV and one compressed sensing (CS) (R = 7.7) accelerated DV 4D flow MRI scans. The same scans were repeated five to ten days after the initial scan in all subjects for interscan reliability assessment. The flip angle was the same (7°) for all scan types. The velocity encoding (venc) value was 160 cm/s for GRAPPA-accelerated SV acquisitions, whereas both low (80 cm/s) and HV (160 cm/s) were used for the DV acquisitions. The venc strategy was symmetric for the SV scans whereas an asymmetric strategy was used for the DV scans. High-venc (HV) (160 cm/s) data from the DV acquisitions were separately analyzed and the results were compared with the other scan types as well. Table 1 summarizes the rest of the scan parameters for each acquisition type. The field of view and in-plane spatial resolution were not constant between the SV and DV acquisitions as low-venc (LV) scan (part of DV) needs stronger gradients for the velocity encoding. Automatic reconstructions on the scanner were performed before the analysis and phase images from the LV and HV data were jointly processed using the Bayesian method to derive velocity for the DV 4D flow MRI acquisitions [24–26]. The HV was calculated as the phase difference between velocity compensated reference set and high velocity encoded set.

### Image Processing and Segmentation

**Figure 1.**
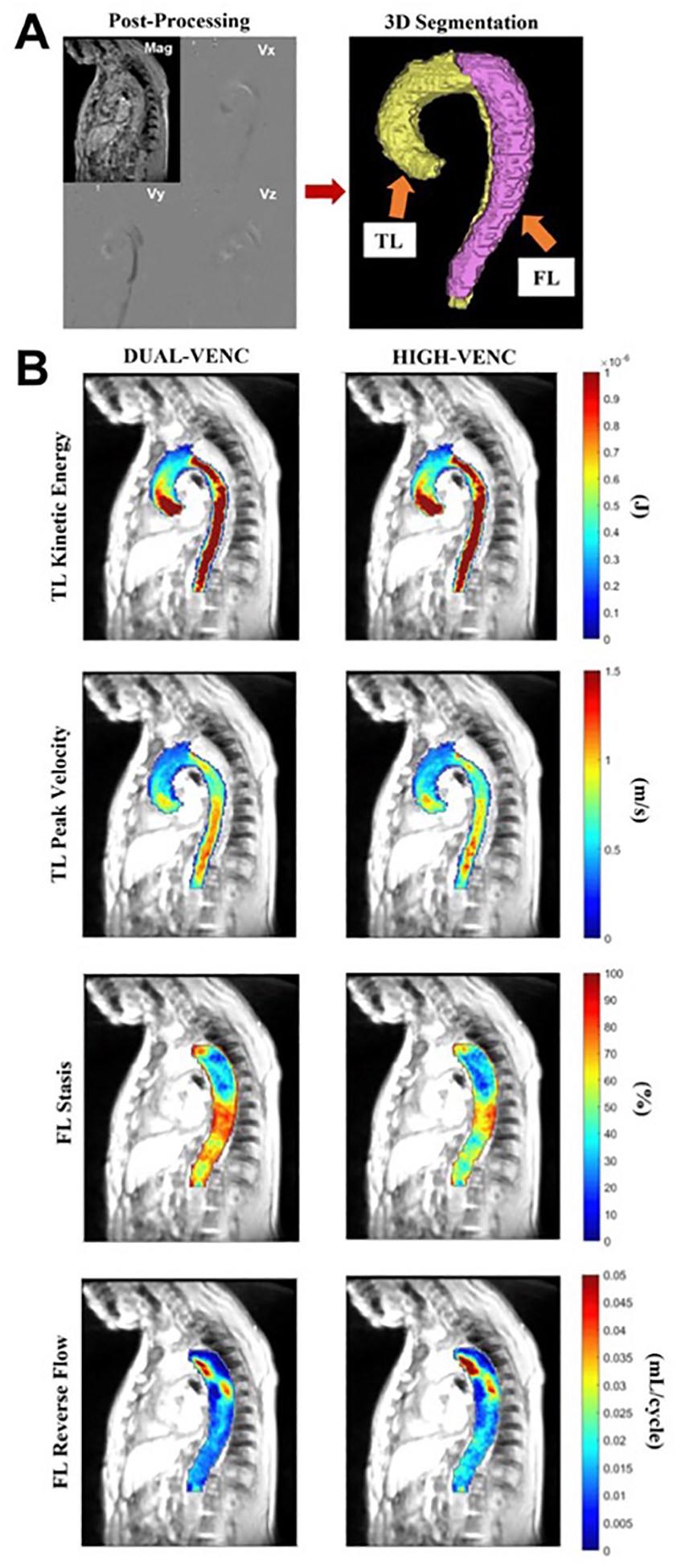
(**A**) Postprocessing of the 4D flow magnetic resonance imaging (MRI) data on MATLAB and manual true (TL) and false lumen (FL) 3D segmentations. (**B**) The volumetric maps of peak velocity and kinetic energy in TL and reverse flow and stasis in FL from the dual-venc (DV) acquisition and high-venc data extracted from the DV acquisition in one subject with de novo type B aortic dissection.

The offline post-processing steps of the 4D flow MRI data were identical for all acquisition types using an in-house tool programmed in Matlab (MATLAB; The MathWorks, Natick, MA) to correct eddy currents, aliasing and noise of the areas outside of the flow regions as described previously [31–33]. Time-averaged magnitude and 3D phase-contrast angiogram (PC-MRA) images were utilized to perform the manual segmentations using the designated software (Mimics Innovation Suite; Materialise, Leuven, Belgium). The entire aorta, excluding aortic arch branch vessels, was manually segmented on the time-averaged 4D flow MRI magnitude images and PC-MRA images were used to segment the TL by an observer (OK) with 3 years of experience in imaging research. The entire aorta was segmented on the PC-MRA images in volunteers and used to mask the 4D flow MRI data. The volunteer data covering the entire aorta were combined with the TL data of the patient group for the TL analysis. The LV and HV data were combined into DV data on the scanner without any additional preprocessing steps. Post-acquisition processing steps, DV acquisition volumetric map examples and the HV data for several TL and FL parameters in one dnTBAD case are displayed in Figure 1.

### Parametric Hemodynamic Maps

The 3D parametric maps for each flow parameter were obtained using in-house analysis tools (MATLAB; The MathWorks, Natick, MA, USA) according to a previously described approach [6,18]. The 4D flow velocity data were interpolated to 1 mm^3^ voxels using spline interpolation. The 3D aortic centerline was calculated automatically based on the TL, and orthogonal planes were automatically placed every millimeter along the centerline. In the next step, each voxel was matched to the closest plane to determine the flow direction along the centerline compared with the normal vector, i.e., forward (ascending aorta to descending aorta) and reverse (descending aorta to ascending aorta). The kinetic energy (KE), forward flow (FF) and 5th percentile peak velocity (PV) were calculated inside the TL and FL for each voxel in the patient group and in the entire aorta of the volunteers. Voxel-wise reverse flow (RF) and stasis were separately calculated in the FL of the patient group, as described in our previous study [31].

#### Forward Flow and Reverse Flow:

The FF and RF were calculated in each voxel through the cardiac cycle and summed. The mean FF and mean RF were reported averaging these sums over the entire volume.

#### Kinetic Energy:

The voxel-wise KE was calculated using the following equation: *KE* = 0.5 *× ρ × dV × v*(*t*)^2^, where the assumed blood density (ρ) was 1060 kg/m^3^, dV the unit voxel volume (i.e., 1 mm^3^) and the velocity magnitude for each voxel at each cardiac timeframe [i.e., v(t)]. The reported KE was calculated as total KE by summing the values in each voxel over the cardiac cycle and then over the entire luminal volume.

#### Peak Velocity:

The 3D PV volumetric maps were determined using the time point with the maximum 95th percentile voxel-wise PV. The mean of the top 5% of velocities was reported as PV for the TL and FL.

#### Stasis:

The voxel-wise flow stasis was defined as the percentage of the cardiac time-frames that the velocity in that voxel is <0.1 m/s. These percentages were averaged over the entire FL volume and reported as mean stasis.

### Statistical Analysis

The repeated measures analysis of variance (ANOVA) method and Pearson correlation coefficients (r) were used to perform groupwise comparisons between the acquisition types. The velocity-noise-ratio (VNR) in the TL in the patient group was calculated by dividing the mean velocity over the entire cardiac cycle in the TL by the velocity noise estimated by the standard deviation (SD) of measured velocities in the static spine. The obtained VNR values were compared pairwise among the acquisition types by the repeated measures ANOVA method. Intraclass correlation coefficients (ICC) were calculated between the baseline and follow-up scans for the interscan reliability assessment of each sequence type using the following reliability levels: <0.5 indicates poor reliability, between 0.5 and 0.75 indicates moderate reliability, between 0.75 and 0.9 indicates good reliability, and any value >0.9 is indicative of excellent reliability [34]. A *p*-value < 0.05 is considered statistically significant. The average scan time and their SDs of the entire cohort consisting of both baseline and follow-up scans were also reported for each acquisition type.

## 3. Results

### Scan Times

The average scan time for GRAPPA-accelerated acquisition is 11.12 +/− 2.64 min and 5.96 +/− 1.33 min for the dual-venc (DV) acquisition in the entire cohort. The scan time reduction by the DV 4D flow magnetic resonance imaging (MRI) acquisition is 46.4% compared with the conventional GRAPPA 4D flow MRI acquisition.

### Hemodynamic 4D Flow MRI Parameters: True Lumen

The mean and standard deviations (SD) of all parameters for all three acquisition types, groupwise comparisons and Pearson correlation coefficients in the true lumen (TL) are summarized in Table 2. Notably, no TL parameters are significantly different among the acquisition types (all *p* > 0.05). The Pearson correlation coefficient analysis demonstrates high correlation (all r > 0.92, all *p* < 0.05) in the TL for all parameters for all pairwise comparisons among the three acquisition types.

### Hemodynamic 4D Flow MRI Parameters: False Lumen

The kinetic energy (KE), peak velocity (PV), forward flow (FF) and stasis are not significantly different between the single-venc (SV) and dual-venc (DV) acquisitions in the false lumen (FL) (*p* > 0.05 for all). The mean reverse flow (RF) is significantly higher in SV acquisition compared with DV acquisition (*p* = 0.009) with a 25% increase of the value in SV acquisition. The KE is significantly higher (*p* = 0.038), and stasis is significantly lower (*p* = 0.01) in high-venc (HV) data compared with SV acquisition. All FL parameters except stasis are significantly higher in HV data compared with DV acquisition (*p* < 0.05 for all) and stasis is significantly lower in HV data compared with DV acquisition (*p* = 0.000).

**Figure 2.**
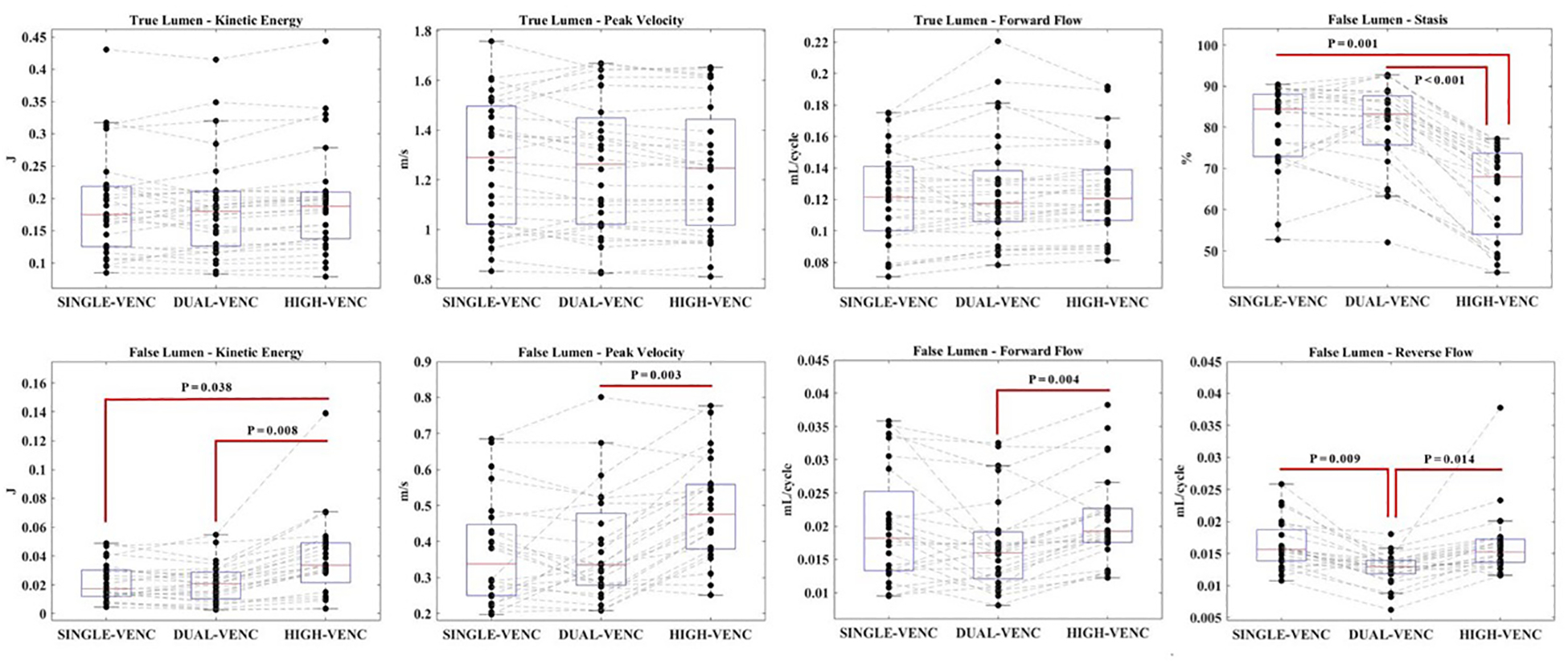
The values for each parameter and their distribution trend are shown in boxplots.

The Pearson correlation coefficient levels in the FL are as follows: r = 0.859, 0.768, 0.751, 0.422 and 0.822 for KE, PV, FF, RF and stasis, respectively, between GRAPPA and DV 4D flow MRI acquisitions (*p* < 0.05 for all); r = 0.636, 0.577, 0.473 and 0.516 for KE, FF, RF and stasis, respectively, between GRAPPA-accelerated 4D flow MRI data and HV data from the DV acquisition with *p* values less than 0.05 for all four correlation levels. On the other hand, r is 0.390 and for PV between GRAPPA and HV data without statistical significance (*p* = 0.590). Pearson correlation coefficient levels between DV and HV data are 0.884, 0.818, 0.869, 0.474 and 0.845 for KE, PV, FF, RF and stasis, respectively (*p* < 0.05 for all parameters). Mean and SDs of all parameters for all three acquisition types, groupwise comparison results and Pearson correlation coefficient levels in the FL are also summarized in Table 2. The values for each parameter and the trend of their distribution among the three acquisition types in TL and FL are represented in Figure 2 boxplots.

### Interscan Reliability Assessment

An excellent level of reliability is observed between baseline and follow-up scans for all three acquisition types for all TL parameters including KE, PV and FF in intraclass correlation coefficient (ICC) analysis with more than 0.931 agreement level for all parameters (*p* < 0.01 for all). The agreement levels in FL are also significantly high for all parameters for SV and DV acquisitions and for KE, stasis, PV and FF in the HV data, (ICC level > 0.800 and *p* < 0.01 for all). The mean RF shows a moderate level of agreement between the first and second scan results in HV data in the FL with a correlation level of 0.640 (*p* = 0.043). The ICC levels between the first and second scans for each dataset are summarized in Table 3.

### Velocity-to-Noise Ratio

The TL velocity-noise-ratio (VNR) value in the patient group for GRAPPA-accelerated acquisition is 3.17 +/− 0.63, 2.82 +/− 1.20 for HV data of the DV 4D flow MRI acquisition and 3.80 +/− 1.39 for the DV 4D flow MRI acquisition. The difference is only significant between the HV and DV acquisitions (HV was 25.7% lower than DV, *p* < 0.001).

## 4. Discussion

In this study we investigate the performance and reliability of compressed-sensing (CS)-accelerated dual-venc (DV) 4D flow magnetic resonance imaging (MRI) in type B aortic dissection (TBAD) compared with the traditional GRAPPA-accelerated 4D flow MRI. Key results include (1) significantly shorter scan times in CS-accelerated DV acquisition, (2) except for the false lumen (FL) reverse flow (RF), there is no significant difference for the true lumen (TL) or FL hemodynamic parameters between DV and single-venc (SV) acquisitions, (3) superior velocity-noise-ratio (VNR) with improved FL hemodynamic quantification using the DV protocol with CS acceleration relative to CS-accelerated high-venc (HV) alone and (4) excellent reproducibility of all three approaches. Our key takeaway is that the VNR gains with CS-accelerated DV acquisitions seem to improve the performance of CS-accelerated DV 4D flow MRI, especially for advanced hemodynamic characterization in regions more predisposed to effects of low VNR such as the FL in TBAD. This feature, combined with the substantial reduction in scan time makes CS-accelerated DV 4D flow MRI an attractive alternative to traditional and standard CS-accelerated 4D flow MRI in disease states with a high dynamic range of velocities such as aortic dissection.

### Hemodynamic parameters in the true lumen and false lumen

It is interesting to note that our results demonstrate no significant difference in any of the hemodynamic TL parameters among three datasets including the conventional GRAPPA-accelerated 4D flow MRI and CS-accelerated DV 4D flow MRI and HV data acquired as a part of the DV acquisition. CS-based reconstruction has been previously used in various studies and underestimation of several 4D flow MRI-derived flow parameters has commonly been observed [13,28–30,35], differently from our study as we do not observe an underestimation of the PV in TL. In their study, Pathrose et al. use three acceleration levels (R = 5.7, 7.7, and 10.2) of CS-accelerated 4D flow MRI protocol in a heterogenous cohort of patients with several aortic disease types and demonstrate an underestimation of several quantitative parameters by all CS based acquisitions. However, the number of TBAD cases is limited to four in their study and the FL analysis results in the dissection cases are not reported separately [13].

On the other hand, FL results demonstrate an overestimation of kinetic energy (KE) and flow parameters peak velocity (PV), forward flow (FF) and RF, and lower estimations of low flow parameter stasis in HV dataset compared with the DV acquisition. As the HV data is a subset of the DV acquisition, these effects are likely secondary to the higher VNR obtained in the DV reconstruction method. Among these hemodynamic parameters quantified, KE is the most unsteady parameter between two datasets with a 90% increase of the mean value from DV data to HV data. The percent change of the PV, FF, RF and stasis are 28.8, 23.5, 33.3 and 19.9%, respectively, which are relatively lower compared with KE. However, these results may still be considered as clinically significant. As KE calculation is directly proportional to the velocity squared, a small number of noisy voxels with a high velocity may be causing the higher average KE values and CS acceleration may be further contributing to the observed higher estimations by increasing the noise in the images. A similar trend for KE and stasis is also observed in the comparison between SV and HV datasets, which is likely secondary to the effects of the CS acceleration. Furthermore, these significant differences in KE and stasis support the idea of the impacts of the KE calculation method besides the impacts of the CS acceleration technique on the image noise. Additionally, the velocities are lower in FL compared with TL which leads to more susceptibility to image noise and velocity encoding (venc)-induced image noise limits the dynamic range of the measurable velocities, making the use of a DV approach in 4D flow MRI acquisitions more important.

### Comparison to previously published dual-venc studies

Schnell et al. use the k-t GRAPPA-accelerated DV 4D flow MRI sequence in sixteen volunteers to evaluate its utility to assess intracranial hemodynamics including net flow and PV. The regional flow quantification in their study demonstrates a similar PV at all arterial locations, as seen in both TL and FL analyses in our study. Net flow analysis demonstrates significantly higher values in both arterial and venous systems in the DV data compared with the HV data. Our results indicate similar FF results between SV and DV acquisitions in both TL and FL, and RF is relatively lower in DV acquisition compared with the SV. The phantom experiments in their study demonstrate 51.4% noise reduction in DV acquisitions compared with HV and the volunteer data shows decreased noise in DV compared with HV [22].

In another study, Schnell et al. acquire k-t GRAPPA-accelerated DV 4D flow MRI with ascending and descending aorta coverage in four Marfan syndrome patients and one bicuspid aortic valve patient and demonstrate an improved VNR in the DV 4D flow MRI data compared with the HV scan and significantly correlated PV results between DV and HV in ascending aorta and arch, as seen in our TL analysis results [20]. The TL VNR results in our patient group maintain the high VNR values in DV acquisition without any significant change compared with SV acquisition. However, as expected, HV data results demonstrate a VNR reduction compared with both DV and SV acquisitions reaching to the significance level only in comparison with DV acquisition. Our results confirm that DV acquisition preserves the favorable VNR of the low-venc (LV) data and the reduction in the VNR in HV data might be related to the CS acceleration which is neutralized by using both LV and HV reconstruction in the DV acquisition. Additionally, descending aorta dissection is the unique pathology in our study, distinctly from the above studies, and CS-acceleration and DV reconstruction methods are investigated in both high- and low-flow environments.

The scan-time reduction by DV 4D flow MRI acquisition is 46.4% compared with the conventional 4D flow MRI in our study, supporting the potential of the CS-accelerated DV 4D flow MRI for clinical translation with significantly shorter scan times, entire velocity dynamic range coverage in the TL and FL avoiding the velocity aliasing and improved VNR relative to the HV acquisition alone. Shorter scan times increase the imaging efficiency and improve patient comfort, which is desirable in critically ill TBAD cases along with the importance of faster imaging for clinicians to take accurate steps in treatment management.

The rigidly set velocity encoding value has been a common issue in 4D flow MRI in various pathologies including aortic dissection. In TBAD, this problem specifically lies in the substantial differences in the blood velocity of TL and FL. However, the measurement of high and low flows in the vessel can be effectively achieved by the advanced Bayesian multipoint velocity encoding method [24–26]. The DV 4D flow MRI technique has been used in various studies representing the dynamic velocity range more accurately in brain and cardiac vessels [22,26,36–38]. However, the effects resulting from the combination of the CS acceleration and DV technique on 4D flow MRI based hemodynamic flow quantifications in TBAD have not been reported in TL and FL separately. Additionally, these 4D flow MRI-derived hemodynamic parameters have not been implemented in diagnostic and/or prognostic criteria in TBAD and neither any gold standard hemodynamic flow quantification method nor any 4D flow MRI based hemodynamic parameter value has been validated as a reference for TBAD patients. Moreover, the fact that no difference is observed between the three datasets in the TL and the same trend is also observed for most parameters in the comparison between SV and DV acquisitions in the FL is promising for future applications of this technique in clinical settings with additional benefits of the CS acceleration scan time savings compared with conventional 4D flow MRI, reliable results as seen in our study between the baseline and follow-up scans with high interscan agreements and high-level image quality similar to the conventional 4D flow MRI. The observed differences in comparisons of the parameters between HV data and both DV and SV acquisitions are likely associated with CS acceleration as similar results were previously observed in comparison between GRAPPA-accelerated and CS-accelerated SV 4D flow MRI acquisitions in the same cohort of patients [31].

Our study has several limitations. The first is the small size of the cohort. Further investigation of the DV acquisition in TBAD in larger cohorts needs to be performed in future studies to establish reference normal values for each hemodynamic parameter with this technique. Even though an increase in the VNR is observed with the DV acquisition compared with the SV acquisition, the observed marginal increase is not statistically significant and may be secondary to the small number of the subjects. The increase in the VNR with the DV acquisition may be better observed in studies with larger cohorts which may better help to assess the clinical correlations and accuracy and precision of the PV and flow measurements. Secondly, the TL and FL segmentations are challenging, especially on non-contrast images. The utilization of the high blood-tissue contrast anatomical imaging registered to flow data or machine-learning based methods may address this issue. Additionally, in cases of actively moving FL, static masks used in the methodology may have impacted the capture of the entire FL through the cardiac time point. Our study is also limited in that we investigate only one LV, HV pairing. However, the HV was chosen so to prevent aliasing while still maximizing the VNR within the aorta. While reducing the LV may increase the VNR in DV 4D flow MRI reconstruction, increasing the gap between the LV and HV may reduce the ability to correctly unwrap the aliased data. Further investigation of the DV acquisition remains needed in future studies to address this problem. Several differences between the acquisition parameters between GRAPPA-accelerated SV acquisitions and CS-accelerated DV acquisitions such as temporal resolution, image orientation and the need for navigator gating in sagittally-acquired DV images, higher signal-to-noise ratio for larger field-of-view in coronally acquired SV images and potential breathing/fat artefacts in the sagittal views may also be the factors behind the reasons of the differences seen. Specifically, temporal resolution of the SV scan vary greatly and more than the DV scan (26–52 ms vs. 36.48 ms) and inferior temporal resolution could be the cause of the surprisingly similar PV estimations. Therefore, future studies matching all scan parameters between the acquisition types may address this limitation. The CS acceleration in the DV acquisition and comparisons of the results with the traditional GRAPPA acceleration and using only a single acceleration level are the other limitations of our study in addition to the scan parameter differences between the acquisition types. Ideally, the only difference between the DV and SV acquisitions would be the venc selections, however the CS acceleration is needed to shorten the scan times in DV acquisitions where the scanner reconstruction times are already longer secondary to the unwrapping. Different CS acceleration levels should also be investigated in future studies.

## 5. Conclusions

In conclusion, our study highlights the potential of dual-venc (DV) acquisitions to substantially reduce scan times and improve the velocity-noise-ratio (VNR), velocity measurements and the quantification of advanced hemodynamic parameters in true lumen (TL) and false lumen (FL) of type B aortic dissection (TBAD) cases with a single 4D flow magnetic resonance imaging (MRI) acquisition in spite of the significant dynamic range of velocities encountered in these patients. Further investigations in larger cohort cohorts are necessary to validate our results and establish reference normal values for quantitative flow parameters with this method.

## Figures and Tables

**Table 1.** 4D flow magnetic resonance imaging (MRI) scan parameters and mean and standard deviation of the scan time for each acquisition type (scan time was not applicable to high-venc 4D flow MRI data as it was derived from the dual-venc 4D flow MRI acquisition).

Acquisition Type	Single-Venc 4D Flow MRI	Dual-Venc 4D Flow MRI	High-Venc 4D Flow MRI
**Scanner**	1.5T MRI	1.5T MRI	1.5T MRI
**Contrast**	No	No	No
**Scan Time (min)**	11.12 +/− 2.64	5.96 +/− 1.33	n/a
**Acceleration Factor (R)**	2	7.7	7.7
**Field of View (mm^2^)**	365–459 × 459–499	306–399 × 380–399	306–399 × 380–399
**Slice Thickness (mm)**	2.8–3.5	2.5–2.8	2.5–2.8
**Repetition Time (ms)**	4.5–6.2	4.5–5.2	4.5–5.2
**Echo Time (ms)**	2.18	3	3
**Spatial Resolution (mm^3^)**	2.6 × 2.6 × 2.8–3.5	2.1–2.2 × 2.1–2.2 × 2.5–2.8	2.1–2.2 × 2.1–2.2 × 2.5–2.8
**Temporal Resolution (ms)**	26.7–52.8	36.5–41.8	36.5–41.8
**Flip Angle (°)**	7	7	7
**Velocity Encoding (cm/s)**	160	80 and 160	160

**Table 2.** Each true and false lumen parameter for single-venc and dual-venc (DV) acquisitions and high-venc data results separately from the DV acquisition, groupwise comparison results and Pearson correlation coefficient levels (* indicates statistical significance).

True Lumen		SINGLE-VENC vs. DUAL-VENC		SINGLE-VENC vs. HIGH-VENC		DUAL-VENC vs. HIGH-VENC	
		SINGLE-VENC	DUAL-VENC	SINGLE-VENC	HIGH-VENC	DUAL-VENC	HICH-VENC
**Total Kinetic Energy (J)**	**Mean ± SD**	0.188 ± 0.079	0.186 ± 0.078	0.188 ± 0.079	0.195 ± 0.083	0.186 ± 0.078	0.195 ± 0.083
	***p* value**	0.053					
	**Correlation**	0.955 *		0.953 *		0.989 *	
**%5 Peak Velocity (m/s)**	**Mean ± SD**	1.267 ± 0.261	1.256 ± 0.267	1.267 ± 0.261	1.240 ± 0.260	1.256 ± 0.267	1.240 ± 0.260
	***p* value**	0.189					
	**Correlation**	0.951 *		0.939 *		0.992 *	
**Mean Fonward Flow (mL/cycle)**	**Mean ± SD**	0.121 ± 0.030	0.126 ± 0.034	0.121 ± 0.030	0.125 ± 0.029	0.126 ± 0.034	0.125 ± 0.029
	***p* value**	0.302					
	**Correlation**	0.919 *		0.935 *		0.975 *	
False Lumen		SINGLE-VENC vs. DUAL-VENC		SINCLE-VENC vs. HIGH-VENC		DUAL-VENC vs. HIGH-VENC	
		SINGLE-VENC	DUAL-VENC	SINGLE-VENC	HIGH-VENC	DUAL-VENC	HIGH-VENC
**Total Kinetic Enengy (J)**	**Mean ± SD**	0.021 ± 0.013	0.020 ± 0.013	0.021 ± 0.013	0.038 ± 0.028	0.020 ± 0.013	0.038 ± 0.028
	***p* value**	0.566		0.038 *		0.008 *	
	**Correlation**	0.859 *		0.636 *		0.884 *	
**%5 Peak Velocity (m/s)**	**Mean ± SD**	0.371 ± 0.148	0.381 ± 0.152	0.371 ± 0.148	0.488 ± 0.140	0.381 ± 0.152	0.488 ± 0.140
	***p* value**	0.626		0.076		0.003 *	
	**Correlation**	0.768 *		0.390		0.818 *	
**Mean Forward Flow (mL/cycle)**	**Mean ± SD**	0.019 ± 0.008	0.017 ± 0.007	0.019 ± 0.008	0.021 ± 0.006	0.017 ± 0.007	0.021 ± 0.006
	***p* value**	0.363		0.388		0.004 *	
	**Correlation**	0.751 *		0.577 *		0.869 *	
**Mean Reverse Flow (mL/cycle)**	**Mean ± SD**	0.016 ± 0.003	0.012 ± 0.002	0.016 ± 0.003	0.016 ± 0.005	0.012 ± 0.002	0.016 ± 0.005
	***p* value**	0.009 *		0.908		0.014 *	
	**Correlation**	0.422 *		0.473 *		0.474 *	
**Mean Stasis (%)**	**Mean ± SD**	80.13 ± 10.39	80.48 ± 10.46	80.13 ± 10.39	64.46 ± 10.99	80.48 ± 10.46	64.46 ± 10.99
	***p* value**	0.784		0.001 *		<0.001 *	
	**Correlation**	0.822 *		0.516 *		0.845 *	

**Table 3.** Intraclass correlations between the baseline and follow-up scans for each acquisition type. Correlation levels for all true lumen and false lumen parameters show statistical significance (*p* < 0.05).

True Lumen		SINGLE-VENC Scan 1 vs. Scan 2	DUAL-VENC Sean 1 vs. Sean 2	HIGH-VENC Scan 1 vs. Scan 2
**Intraclass Correlation levels**	**Kinetic Energy (J)**	0.948	0.931	0.949
	**Peak Velocity (m/s)**	0.957	0.965	0.958
	**Forward Flow (mL/cycle)**	0.973	0.945	0.935
False Lumen		SINGLE-VENC Scan 1 vs. Scan 2	DUAL-VENC Scan 1 vs. Scan 2	HIGH-VENC Scan 1 vs. Scan 2
**Intraclass Correlation Levels**	**Kinetic Energy (J)**	0.983	0.890	0.805
	**Reverse Flow (mL/cycle)**	0.907	0.851	0.640
	**Peak Velocity (m/s)**	0.989	0.926	0.925
	**Forward Flow (mL/cycle)**	0.975	0.970	0.882
	**Stasis (%)**	0.974	0.924	0.851

## Data Availability

The data that support the findings of this study are available on request from the corresponding author, O.K. The data are not publicly available due to data sets containing information that could compromise research participant privacy.

## Abbreviations

ANOVA: Analysis of variance
CS: Compressed sensing
CTA: Computed tomography angiography
dnTBAD: De novo Type B aortic dissection
DV: Dual-venc
FF: Forward flow
FL: False lumen
HV: High-venc
ICC: Intraclass correlation coefficient
KE: Kinetic energy
MRA: Magnetic resonance angiography
MRI: Magnetic resonance imaging
PC-MRA: Time-averaged 3D phase contrast magnetic resonance angiogram
PV: Peak velocity
R: Acceleration factor
RF: Reverse flow
rTAAD: repaired TAAD with residual TBAD
SD: Standard deviation
SV: Single-venc
TAAD: Type A aortic dissection
TBAD: Type B aortic dissection
TL: True lumen
v: velocity
Venc: Velocity encoding
VNR: Velocity-to-noise ratio
